# Diabetic retinopathy severity is associated with renal function deterioration in patients with diabetic kidney disease: a retrospective cohort study

**DOI:** 10.3389/fendo.2026.1757296

**Published:** 2026-02-26

**Authors:** Liping Yu, Yanrong Zhang, Chuan Sun, Haipeng Zhao, Yujie Yan

**Affiliations:** 1 Endocrinology Department, China-Japan Friendship Hospital, Beijing, China; 2 Endocrinology Department, The Second Hospital of Shijiazhuang, Hebei, China; 3 Ophthalmology Department, China-Japan Friendship Hospital, Beijing, China

**Keywords:** diabetic kidney disease, diabetic retinopathy, estimated glomerular filtration, hard exudates, renal function deterioration

## Abstract

**Background:**

To investigate whether the severity of diabetic retinopathy (DR) and the presence of hard exudates (HEs) are associated with renal function deterioration in patients with diabetic kidney disease (DKD).

**Methods:**

This is a retrospective cohort study including 140 patients with DKD. The outcome was the progression of DKD (an estimated glomerular filtration (eGFR) decline (%)>15%) over a 5-year follow-up period. A total of 101 patients had eGFR parameters during the follow-up. DR was categorized into nonproliferative DR (NPDR) and proliferative DR (PDR). HEs were identified via optical coherence tomography (OCT). Clinical and laboratory data were acquired from medical records. The influence of the severity of DR and the presence of HEs were assessed via Cox regression.

**Results:**

The mean follow-up time was 34.31 (± 16.36) months. A significant difference was found in eGFR decline (%) (P = 0.024) between the absent DR, NPDR and PDR groups. eGFR decline (%) was more severe in patients with HEs than in those without HEs (P = 0.011). After adjustment for age, body mass index (BMI), glycosylated hemoglobin (HbA1c), low-density lipoprotein cholesterol (LDL-C), systolic blood pressure, eGFR at baseline, urine albumin creatine ratio (UACR) stage at baseline, use of Sodium-Glucose Linked Transporter 2 (SGLT2) inhibitors, as well as potential confounders such as duration of Diabetic mellitus (DM), use of Renin-Angiotensin System Inhibitors (RAS) inhibitors and Glucagon-Like Peptide-1 Receptor Agonists (GLP-1RAs), Cox regression revealed that PDR (p=0.035) and NPDR (p=0.049) were independently associated with renal function deterioration. Compared with the participants in the absent DR group, participants with PDR, as well as NPDR presented a nearly threefold greater risk (adjusted HR = 2.88; 95% CI: 1.08–7.71; adjusted HR = 2.78; 95% CI: 1.004–7.70, respectively). However, the presence of HEs was not independently associated with renal function deterioration in the adjusted Cox model (P = 0.567).

**Conclusions:**

DR severity was independently associated with the progression of DKD, whereas HEs were not. DKD patients with PDR as well as NPDR should undergo kidney function testing more frequently and receive early intervention to prevent renal function deterioration.

## Introduction

Diabetic mellitus (DM) has become a critical global health issue over the past few decades and is characterized by high prevalence rates and significant morbidity with complications (1–4). In 2021, 537 million adults were living with diabetes worldwide, as reported by the World Health Organization (WHO) and the International Diabetes Federation (IDF). This number is set to 783 million by 2045, placing considerable strain on global healthcare systems (5).

The microvascular complications associated with DM include diabetic retinopathy (DR) and diabetic kidney disease (DKD), which share similar pathophysiological pathways and significantly impact patients’ quality of life (6). Previous studies have established a significant association between DR and renal function decline. The incidence of DR is greater in patients with DKD than in those without DKD (7–12). Furthermore, the effects of DR and DKD extend beyond vision-threatening and renal outcomes (13–16). In a multiethnic population-based cohort study including 2964 Asian adults, DR and DKD were independently associated with increased risks of all-cause and cardiovascular mortality. These findings highlight the importance of early identification, close monitoring and effective management of patients with DR and DKD to mitigate the risk of mortality (13). Therefore, understanding the connection between DR and DKD, along with vigilant monitoring of these microvascular complications, is crucial.

Hard exudates (HEs) result from the leakage and accumulation of lipids and lipoproteins from incompetent microaneurysms and capillaries into the retinal layers, making them easily visible clinical markers of microvascular damage in the retina (17). They arise from the compromised integrity of the blood–retinal barrier and inflammatory processes triggered by the diabetic state, which are also associated with renal function impairment and the formation of urinary albumin. The disappearance of diabetic macular HEs has been documented after patients undergo hemodialysis to treat chronic renal failure secondary to DKD (18), suggesting that renal dysfunction and retinal protein leakage may occur in parallel. Furthermore, our previous study (19)revealed a significant difference in the urinary albumin–creatinine ratio (UACR) between DKD patients with and without HEs, highlighting the potential utility of HEs as a prognostic indicator for renal function.

This study therefore aimed to investigate the associations between the severity of DR, as well as the presence of HEs, and renal function progression in patients with DKD. This study aims to provide evidence of DR severity and HEs as indicators of renal microvascular conditions and to offer clinicians a reliable method to assess renal risk on the basis of ophthalmic examination.

## Methods

This was a retrospective observational cohort study. A total of 140 in patients who were diagnosed with type 2 DM and DKD and who had comprehensive ophthalmic examinations were enrolled from the China-Japan Friendship Hospital between January and December 2019 to serve as the baseline population. The diagnosis of DKD was primarily based on the following two key findings, which persisted for more than 3 months (1): albuminuria, a raised urine albumin-to-creatinine ratio (UACR), specifically a UACR ≥ 30 mg/g; and (2) reduced kidney function, a decreased estimated glomerular filtration rate (eGFR), specifically an eGFR < 60 mL/min/1.73 m². The presence of either criterion in a person with diabetes established the diagnosis, with the absence of signs indicating other kidney diseases. The medical records of these patients were reviewed until January 2024, and the most recent laboratory data, along with the corresponding testing dates, were recorded. The aforementioned testing date was defined as the final follow-up data. Ultimately, 101 patients underwent eGFR level testing during the follow-up period. Ethical approval for this study was obtained from the Institutional Review Board of the China-Japan Friendship Hospital. The study was conducted in full adherence to the tenets of the Declaration of Helsinki.

The clinical and laboratory data were extracted from medical records at both baseline and the final visit. The collected details included key clinical and demographic variables, including age, sex, diabetes duration, body mass index (BMI), abdominal circumference, hypertension history, blood pressure, glycosylated hemoglobin (HbA1c) level, serum lipid levels, estimated glomerular filtration rate (eGFR) and urine albumin creatine ratio (UACR). Use of antihypertensive medication, such as Renin-Angiotensin System (RAS) inhibitors, and glucose-lowering medications, such as Sodium-Glucose Linked Transporter 2 (SGLT2) inhibitors and Glucagon-Like Peptide-1 Receptor Agonists (GLP-1RAs) were also collected. The eGFR was calculated via established formulas on the basis of serum creatinine (Scr), age, and sex. The eGFR was calculated via the Chronic Kidney Disease Epidemiology Collaboration (CKD-EPI) creatinine equation (2009). The formula is as follows:


$$\text{For men},\text{if Scr}\leq 0.9\text{ mg}/\text{dl},\text{ eGFR }=\;141\;\times \;{\left(\text{Scr}/0.9\right)}^{-0.411}\times 0.993^{\text{age}}$$

$$\text{if Scr }>0.9\text{ mg}/\text{dl},\text{ eGFR }=\;141\;\times \;{\left(\text{Scr}/0.9\right)}^{-1.209}\times 0.993^{\text{age}}$$

$$\text{For women},\text{if Scr}\leq 0.7\text{ mg}/\text{dl},\text{ eGFR }=\;144\;\times \;{\left(\text{Scr}/0.7\right)}^{-0.329}\times 0.993^{\text{age}}$$

$$\text{if Scr }>0.7\text{ mg}/\text{dl},\text{ eGFR }=\;144\;\times \;{\left(\text{Scr}/0.7\right)}^{-1.209}\times 0.993^{\text{age}}$$

The results are expressed in mL/min/1.73 m², where Scr is expressed in mg/dl and age is expressed in years.

A comprehensive ophthalmic evaluation was performed at baseline, encompassing best-corrected visual acuity (BCVA) assessment with a standardized Snellen chart, intraocular pressure (IOP) measurement via applanation tonometry, slit-lamp biomicroscopy, color fundus photography, optical coherence tomography (OCT), and fluorescein angiography (FA) when necessary. Diabetic retinopathy (DR) stage was initially determined by general ophthalmologists through systematic analysis of color fundus images, OCT scans, and FA findings. To ensure diagnostic consistency, all evaluations were subsequently verified by an experienced retinal specialist (author YYJ). Diabetic retinopathy (DR) stages were classified according to established criteria (20) as follows (1): non-DR: absence of any visible retinopathic signs (2); nonproliferative DR (NPDR): presence of retinal abnormalities, such as microaneurysms, hemorrhages, hard exudates (HEs), or cotton-wool spots, in the absence of PDR features; and (3) proliferative DR (PDR): characterized by definite neovascularization, vitreous/preretinal hemorrhage, or tractional retinal detachment. For each patient, the eye with the most advanced stage of DR was selected for analysis. Hard exudates (HEs) were identified as well-defined, white-yellow deposits located in the posterior pole upon fundus examination. The presence of HEs was further confirmed via cross-sectional optical coherence tomography (OCT), which typically reveals HEs as discrete, hyperreflective foci within the outer plexiform layer.

The primary outcome was the progression of DKD, which was defined by the percentage decrease in the eGFR at the last visit. The eGFR decline(%) was calculated via the following formula: eGFR decline (%) = [(eGFR at baseline – eGFR at the last visit)/eGFR at baseline] *100 (%). The median eGFR decline (%) was established as 15%. Consequently, the progression of DKD was defined as an eGFR decline (%) greater than 15% compared with baseline values.

### Statistics

Statistical analyses were performed via SPSS (version 26.0; IBM Corp., Armonk, NY, USA) and SAS 9.4 (SAS Institute). Continuous data are presented as the mean ± standard deviation (SD) if normally distributed or as the median and interquartile range (IQR) if nonnormally distributed. Categorical variables are expressed as numbers and percentages (n, %). Comparisons between groups were conducted via appropriate tests: Student’s *t*-test or one-way ANOVA for normally distributed continuous variables and the Mann–Whitney U test or Kruskal–Wallis test for nonnormally distributed variables. Associations between categorical variables were assessed via the chi-square test or Fisher’s exact test, as applicable. Fully Conditional Specification (FCS) approach in SAS (PROC MI) was used to impute the 27 missing values in UACR baseline. To identify independent predictors associated with DKD progression, Cox regression analysis and multivariate logistic regression analysis were employed. A two-sided P value of < 0.05 was defined as the threshold for statistical significance.

## Results

The medical records of 140 patients with type 2 DM and DKD who underwent comprehensive ophthalmic examinations were reviewed. Among these cases, the absence of DR accounted for 38.6%, NPDR accounted for 33.6%, and PDR accounted for 27.9%. Clinical parameters were collected at baseline and at the last visit over a five-year follow-up period. Among the participants, 101 patients had available eGFR parameters during the follow-up period. 39 patients lost follow-up because of far-away living (30 patients), unwillingness to come (3 patients) and lost contact with (6 patients). The mean follow-up time was 34.31 (± 16.36) months.

**Figure 1:**
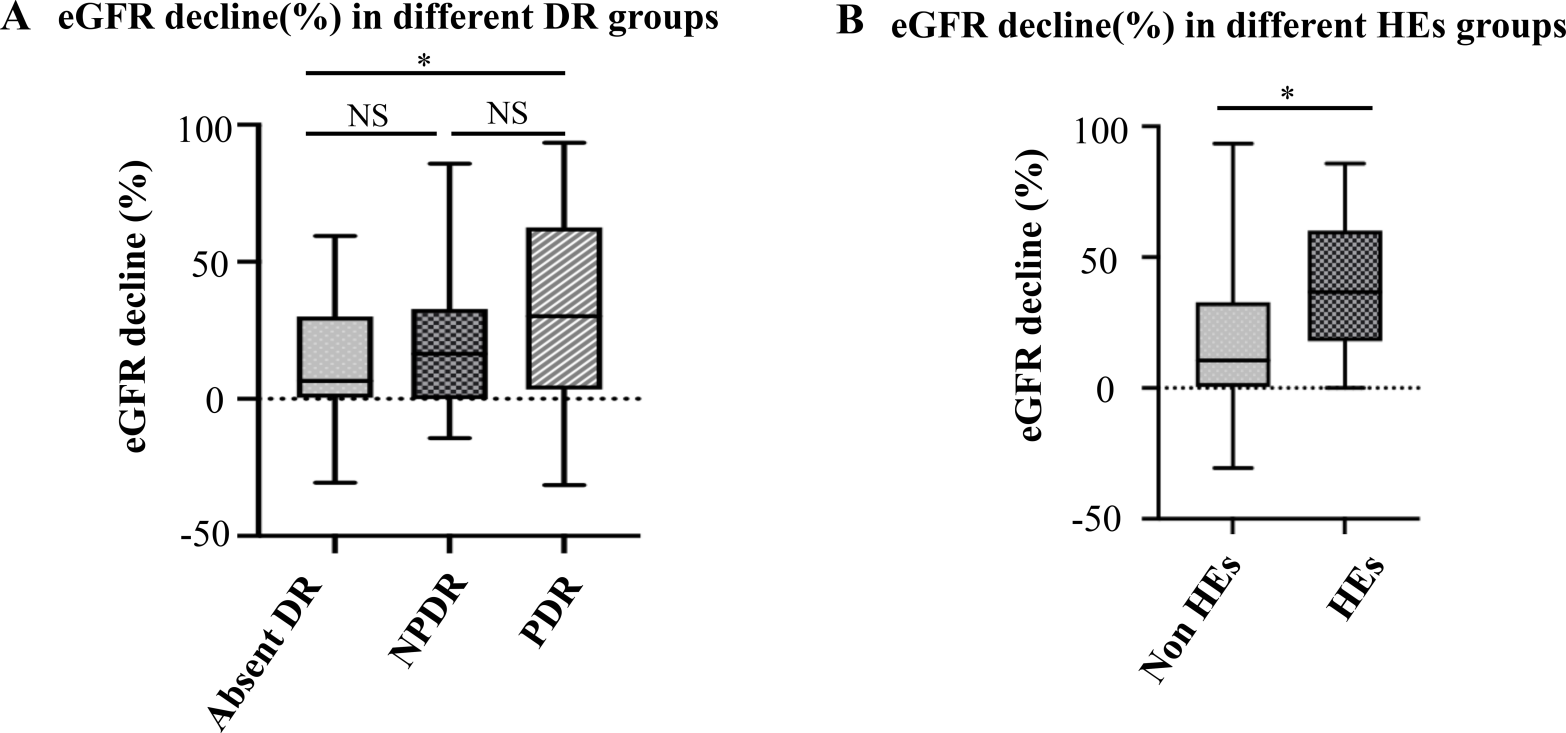
eGFR decline (%) in different DR groups **(A)** and HEs groups **(B)**. **(A)** The eGFR decline was 6.52 (0.38, 30.02) % in the absent DR group, 16.50 (0.08, 32.84) % in the NPDR group and 30.22 (3.39, 62.50) % in PDR group. Significant difference was found between absent DR group and PDR group (P = 0.007), but not found between absent DR group and NPDR group (P = 0.450), as well as NPDR group and PDR group (P = 0.075). **(B)** The eGFR decline was 10.54 (1.23, 33.32) % in the non-HEs group and 20.69 (4.10, 51.39) % in HEs group. Significant difference was found between these two groups (P = 0.011). *p value <0.05. eGFR, Estimated glomerular filtration rate; DR, Diabetic retinopathy; NPDR, Non-proliferative diabetic retinopathy; PDR, Proliferative diabetic retinopathy; HEs, Hard exudates.

There were no significant differences among the absent DR group, NPDR group and PDR group in terms of sex, age, BMI, duration of DM, HbA1c level, blood pressure or serum lipid profile at baseline or follow-up. However, visual acuity (P<0.001), eGFR (P<0.001) and UACR stage (P<0.001) were significantly different at baseline. While there were no significant differences in follow-up time, the eGFR decline (%) was significantly different among the three groups (P = 0.024), as presented in **Table 1**. The eGFR decline (%) in the PDR group was significantly more pronounced than that in the non-DR group. In contrast, no significant difference was noted in the decrease in the eGFR (%) between the NPDR group and the non-DR group (**Figure 1A**).

**Table 1:** Demographic and clinical characteristics in absent-DR, NPDR and PDR groups.

Variable	Absent-DR	NPDR	PDR	P
Baseline	N=54	N=47	N=39	N=140
Gender N (%) 0.087				
Male	39 (45.3)	24 (27.9)	23 (26.7)	
Female	15 (27.8)	23 (42.6)	16 (29.6)	
Age(years)	62.65 (± 13.14)	58 21(± 12.36)	59.46(± 12.01)	0.191
BMI (kg/m^2)	26.74 (± 4.36)	28.84 (± 3.81)	26.56 (± 3.74)	0.507
Abdominal circumference(cm)	98.29 (± 12.59)	92.54(± 10.43)	97.70(± 14.43)	0.134
Duration of DM (years)	14.00 (8.00,20.00)	13.00 (8.00,17.00)	20.00 (10.00,21.00)	0.087
HbA1c (%)	8.25 (7.10,9.70)	9.65 (7.70,10.68)	8.15 (7.15,10.28)	0.086
History of hypertension N (%)				
No	12 (44.4)	9 (33.3)	6 (22.2)	0.743
Yes	42 (37.5)	38 (33.9)	32 (28.6)	
Systolic pressure (mmHg)	137.50 (120.00,152.25)	147.00 (126.00,156.00)	143.00 (127.00,170.75)	0.179
Diastolic pressure (mmHg)	77.67 (± 11.23)	79.94 (± 10.19)	81.66 (± 12.87)	0.245
Serum lipid profiles				
Total cholesterol (mmol/l)	4.04(3.51, 4.56)	4.24(3.58, 5.32)	4.37(3.13, 5.74)	0.242
Triglycerides (mmol/l)	1.84(1.39, 2.81)	1.89(1.21, 2.75)	1.63(1.19, 2.51)	0.654
HDL cholesterol (mmol/l)	0.96(0.83, 1.12)	0.98(0.89, 1.19)	1.04(0.88, 1.17)	0.327
LDL cholesterol (mmol/l)	2.56(1.96, 2.83)	2.68(2.12, 3.40)	2.78(1.82, 3.52)	0.086
eGFR baseline (ml/min/1.73m^2)	89.60 (58.76, 102.45)	83.71 (60.42,100.34)	57.51 (37.38,84.30)	<0.001^*^
UACR stage baseline				
UACR stage 1(<300mg/g)	37 (53.6)	21 (30.4)	11 (16.0)	<0.001^*^
UACR stage 2(≥300mg/g)	17 (23.9)	26 (36.6)	28 (39.4)	
Visual acuity (LogMAR)	0.80(0.50, 0.95)	0.50(0.30, 0.80)	0.50(0.20, 0.60)	<0.001^*^
Use of RAS inhibitors N (%)				
No	39 (37.5)	35 (33.7)	30 (28.8)	0.832
Yes	15 (41.7)	12 (33.3)	9 (25.0)	
Use of SGLT2 inhibitors N (%)				
No	42 (36.8)	37 (32.5)	35 (30.7)	0.286
Yes	12 (46.2)	10 (38.5)	4 (15.4)	
Use of GLP-1RAs N (%)				
No	50 (37.3)	45 (33.6)	39 (29.1)	0.242
Yes	4 (66.7)	2 (33.3)	0 (0.0)	
Follow-up	N=41	N=28	N=29	N=98^#^
Follow-up Time (months)	36.66 (± 18.94)	34.00 (± 15.17)	33.03 (± 13.67)	0.628
HbA1c (%)	7.25 (6.68,8.60)	8.00 (6.70,10.33)	7.50 (6.20,8.80)	0.499
Systolic pressure (mmHg)	135.00 (129.25,146.25)	137.00 (125.00,153.50)	150.00 (124.00,182.30)	0.316
Diastolic pressure (mmHg)	79.92 (± 9.09)	77.76 (± 10.12)	76.11 (± 12.04)	0.475
Serum lipid profiles during follow-up				
Total cholesterol (mmol/l)	4.08(3.21, 4.85)	4.24(3.11, 5.20)	4.31(3.56, 5.35)	0.501
Triglycerides (mmol/l)	1.99(1.48, 3.04)	1.75(1.10, 2.35)	1.70(1.05, 2.06)	0.233
HDL cholesterol (mmol/l)	1.05(0.85, 1.20)	1.10(0.97, 1.29)	1.06(0.89, 1.25)	0.571
LDL cholesterol (mmol/l)	2.58(1.80, 2.91)	2.73(1.80, 3.42)	2.77(2.17, 3.45)	0.184
eGFR follow up (ml/min/1.73m^2)	76.21(39.61, 96.92)	57.56(31.96, 87.25)	30.52(12.07, 54.49)	0.005^*^
eGFR decline (ml/min/1.73m^2)	6.81(0.44, 16.82)	9.09(0.08, 23.35)	14.57(3.48, 30.62)	0.095
eGFR decline (%)	6.52(0.38, 30.02)	16.50(0.08, 32.84)	30.22(3.39, 62.50)	0.024^*^

Data are means ± SD, n (%), or median (interquartile range). Comparisons between groups were conducted using appropriate tests: one-way ANOVA for normally distributed continuous variables, and Kruskal-Wallis test for non-normally distributed variables. Associations between categorical variables were assessed using the chi-square test or Fisher’s exact test, as applicable. *p value <0.05.

DM, Diabetic mellitus; DR, Diabetic retinopathy; NPDR, Non-proliferative diabetic retinopathy; PDR, Proliferative diabetic retinopathy; BMI, Body Mass Index; HDL, High-density lipoprotein cholesterol; LDL, Low-density lipoprotein cholesterol; eGFR, Estimated glomerular filtration rate; UACR, Urine albumin creatine ratio; RAS, Renin-Angiotensin System; SGLT2, Sodium-Glucose Linked Transporter 2; GLP-1RAs, Glucagon-Like Peptide-1 Receptor Agonists.

^#^3 patients missing DR stage data among the 101 patients who underwent eGFR level testing during the follow-up period.

A total of 138 eyes from 138 patients with clear color fundus and cross-sectional OCT images were analyzed for the presence of HEs. Among these patients, 40 (29.0%) presented with HEs in the posterior pole region. Patients with HEs demonstrated poorer visual acuity, higher total cholesterol levels, and higher low-density lipoprotein cholesterol (LDL-C) levels than did those without HEs (p=0.010, p=0.009, and p=0.007, respectively) (**Table 2**). There were significant differences in baseline UACR stage (p=0.009), but no significant differences were observed in the baseline eGFR levels and follow-up times. However, eGFR decline (%) was more pronounced in patients with HEs (P = 0.011) (**Figure 1B**, **Table 2**).

**Table 2:** Demographic and clinical characteristics in HEs and non-HEs groups.

Variable	Non-HEs	HEs	P
Baseline	N=98	N=40	N=138
Gender N (%) 0.010^*^			
Male	67(78.8)	18 (21.2)	
Female	31 (58.5)	22 (41.5)	
Age(years)	61.50 (± 12.39)	57.45(± 13.04)	0.880
BMI (kg/m^2)	26.45(23.88, 28.70)	25.05(23.24, 28.00)	0.261
Abdominal circumference(cm)	95.75(89.00, 103.00)	92.00(83.30, 102.40)	0.205
History of hypertension N (%)			
No	19 (70.4)	8 (29.6)	0.956
Yes	78 (70.9)	32 (29.1)	
Systolic pressure (mmHg)	141.00 (125.00,157.00)	148.00 (126.25,163.00)	0.332
Diastolic pressure (mmHg)	80.19 (± 11.61)	78.00 (± 10.90)	0.310
Duration of DM (years)	15.00(8.00, 20.00)	13.00(10.00, 19.50)	0.553
HbA1c (%)	8.25(7.23, 9.90)	9.75(7.68, 10.63)	0.124
Serum lipid profiles			
Total cholesterol (mmol/l)	4.06(3.18, 4.79)	4.48(3.95, 5.44)	0.009^*^
Triglycerides (mmol/l)	1.67(1.20, 2.60)	1.89(1.21, 2.83)	0.665
HDL cholesterol (mmol/l)	0.98(0.85, 1.11)	1.08(0.89, 1.22)	0.113
LDL cholesterol (mmol/l)	2.52(1.86, 2.96)	2.93(2.37, 3.48)	0.007^*^
eGFR baseline (ml/min/1.73m^2)	85.16(56.43, 100.40)	72.49(52.57, 93.15)	0.097
UACR stage baseline			
UACR stage 1(ACR<300mg/g)	56 (81.2)	13 (18.8)	0.009^*^
UACR stage 2(ACR≥300mg/g)	42 (60.9)	27 (39.1)	
Visual acuity (LogMAR)	0.60(0.50, 0.80)	0.40(0.35, 0.60)	0.010^*^
Use of RAS inhibitors N (%)			
No	70 (71.4)	32 (28.6)	0.206
Yes	28 (80.0)	8 (20.0)	
Use of SGLT2 inhibitors N (%)			
No	77 (78.6)	35 (21.4)	0.165
Yes	21 (87.5)	5 (12.5)	
Use of GLP-1Ras N (%)			
No	92 (69.7)	40 (30.3)	0.123
Yes	6 (100.0)	0 (0.0)	
Follow-up	N=71	N =25	N=96^#^
Follow-up Time (months)	36.24 (± 16.66)	33.16 (± 15.04)	0.418
HbA1c (%)	7.30(6.60, 8.70)	8.15(6.70, 10.63)	0.235
Systolic pressure (mmHg)	137.00(126.00, 150.00)	143.00(125.00, 165.00)	0.353
Diastolic pressure (mmHg)	77.77 (± 9.96)	78.41 (± 11.04)	0.824
Serum lipid profiles during follow-ups			
Total cholesterol (mmol/l)	4.17(3.20, 4.86)	4.90(3.67, 5.84)	0.053
Triglycerides (mmol/l)	1.82(1.23, 2.53)	1.91(0.99, 2.45)	0.725
HDL cholesterol (mmol/l)	1.05(0.89, 1.92)	1.15(0.96, 1.51)	0.085
LDL cholesterol (mmol/l)	2.60(1.80, 2.97)	2.95(2.18, 3.58)	0.041^*^
eGFR follow up (ml/min/1.73m^2)	60.40(33.84, 91.88)	40.62(12.24, 59.69)	0.011^*^
eGFR decline (ml/min/1.73m^2)	7.09(1.28, 19.78)	13.19(1.47, 26.14)	0.270
eGFR decline (%)	10.54(1.23, 33.32)	20.69(4.10, 51.39)	0.011^*^

Data are means ± SD, n (%), or median (interquartile range). Comparisons between groups were conducted using appropriate tests: the student’s *t*-test for normally distributed continuous variables, and the Mann-Whitney U for non-normally distributed variables. Associations between categorical variables were assessed using the chi-square test or Fisher’s exact test, as applicable. *p value <0.05.

HEs, Hard exudates; BMI, Body Mass Index; HDL, High-density lipoprotein cholesterol; LDL, Low-density lipoprotein cholesterol, eGFR, Estimated glomerular filtration rate; UACR, Urine albumin creatine ratio; RAS, Renin-Angiotensin System; SGLT2, Sodium-Glucose Linked Transporter 2; GLP-1RAs, Glucagon-Like Peptide-1 Receptor Agonists.

^#^5 patients missing HEs data among the 101 patients who underwent eGFR level testing during the follow-up period.

Among the 101 patients tested for eGFR decline (%), there were no significant differences in sex, duration of DM, follow-up time or blood pressure between the eGFR decline (%) <=15% group and the eGFR decline (%)>15% group. However, significant differences were observed in DR severity (p=0.024), the presence of HEs (p=0.011), visual acuity (p<0.001), age (p=0.017), BMI (p=0.027), the HbA1c level (p=0.017), eGFR level at baseline (p<0.001), UACR stage at baseline (P = 0.002) and use of SGLT2 inhibitors (p=0.011) between the two groups. Significant differences in serum lipid profiles, such as total cholesterol levels (p<0.001) and low-density lipoprotein cholesterol (LDL-C) levels (p<0.001), were also detected between the eGFR decline (%) <=15% and eGFR decline (%) >15% groups (**Table 3**).

**Table 3:** Demographic and clinical characteristics according to progression of DKD.

Variable	eGFR decline (%) <=15	eGFR decline (%) >15	P
	N=50	N=51	N=101
Gender N (%) 0.181			
Male	33 (55.0)	27 (45.0)	
Female	17 (41.5)	24 (58.5)	
Age(years)	58.38 (± 12.89)	64.10(± 10.60)	0.017^*^
BMI (kg/m^2)	27.50(24.72, 29.39)	24.95(23.31, 27.88)	0.027^*^
Abdominal circumference(cm)	98.27 (± 11.02)	93.71(± 13.07)	0.143
Duration of DM (years)	14.90 (± 7.98)	17.55 (± 9.12)	0.128
HbA1c (%)	8.00 (7.25,9.40)	9.60 (7.55,10.80)	0.017^*^
DR status at baseline 0.024^*^			
Non-DR	27 (65.9)	14 (34.1)	
NPDR	12 (42.9)	16 (57.1)	
PDR	10 (34.5)	19 (65.5)	
HEs status at baseline 0.011^*^			
Non-HEs	41(57.7)	30 (42.3)	
HEs	7 (28.0)	18 (72.0)	
History of hypertension N (%) 0.963			
No	9 (50.0)	9 (50.0)	
Yes	41 (49.4)	42 (50.6)	
Systolic pressure (mmHg)	138.16 (± 21.37)	146.16 (± 24.23)	0.082
Diastolic pressure (mmHg)	78.34 (± 11.68)	77.69 (± 10.33)	0.766
Serum lipid profiles			
Total cholesterol (mmol/l)	3.58(2.81, 3.38)	4.44(3.86, 5.79)	<0.001^*^
Triglycerides (mmol/l)	1.74(1.18, 2.68)	2.06(1.22, 2.83)	0.347
HDL cholesterol (mmol/l)	0.92(0.82, 1.07)	1.05(0.87, 1.23)	0.014^*^
LDL cholesterol (mmol/l)	2.17(1.70, 2.79)	2.76(2.41, 3.54)	<0.001^*^
Visual acuity (LogMAR)	0.80(0.55, 0.90)	0.50(0.25, 0.75)	<0.001^*^
eGFR base (ml/min/1.73m^2)	90.04(58.03, 102.29)	58.61(40.91, 78.94)	<0.001^*^
UACR stage baseline			
UACR stage 1(ACR<300mg/g)	30 (66.7)	15 (33.3)	0.002^*^
UACR stage 2(ACR≥300mg/g)	20 (35.7)	36 (64.3)	
Use of RAS inhibitors N (%)			
No	35(54.7)	29 (45.3)	0.171
Yes	15 (40.5)	22 (59.5)	
Use of SGLT2 inhibitors N (%)			
No	31 (41.9)	43 (58.1)	0.011^*^
Yes	19 (70.4)	8 (29.6)	
Use of GLP-1Ras N (%)			
No	45 (47.4)	50 (52.6)	0.112
Yes	5 (83.3)	1 (16.7)	
eGFR follow up (ml/min/1.73m^2)	88.47(58.58, 99.29)	33.73(19.88, 57.55)	<0.001^*^
eGFR decline (ml/min/1.73m^2)	1.26(1.14, 4.21)	21.35(13.60, 32.41)	<0.001^*^
eGFR decline (%)	1.18(3.27, 6.03)	33.82(25.06, 56.87)	<0.001^*^

Data are means ± SD, n (%), or median (interquartile range). Comparisons between groups were conducted using appropriate tests: the student’s *t*-test for normally distributed continuous variables, and the Mann-Whitney U for non-normally distributed variables. Associations between categorical variables were assessed using the chi-square test. *p value <0.05.

DKD, Diabetic kidney disease; DM, Diabetic mellitus; DR, Diabetic retinopathy; NPDR, Non-proliferative diabetic retinopathy; PDR, Proliferative diabetic retinopathy; HEs, Hard exudates; BMI, Body Mass Index; HDL, High-density lipoprotein cholesterol; LDL, Low-density lipoprotein cholesterol, eGFR, Estimated glomerular filtration rate; UACR, Urine albumin creatine ratio; RAS, Renin-Angiotensin System; SGLT2, Sodium-Glucose Linked Transporter 2; GLP-1RAs, Glucagon-Like Peptide-1 Receptor Agonists.

**Figure 2:**
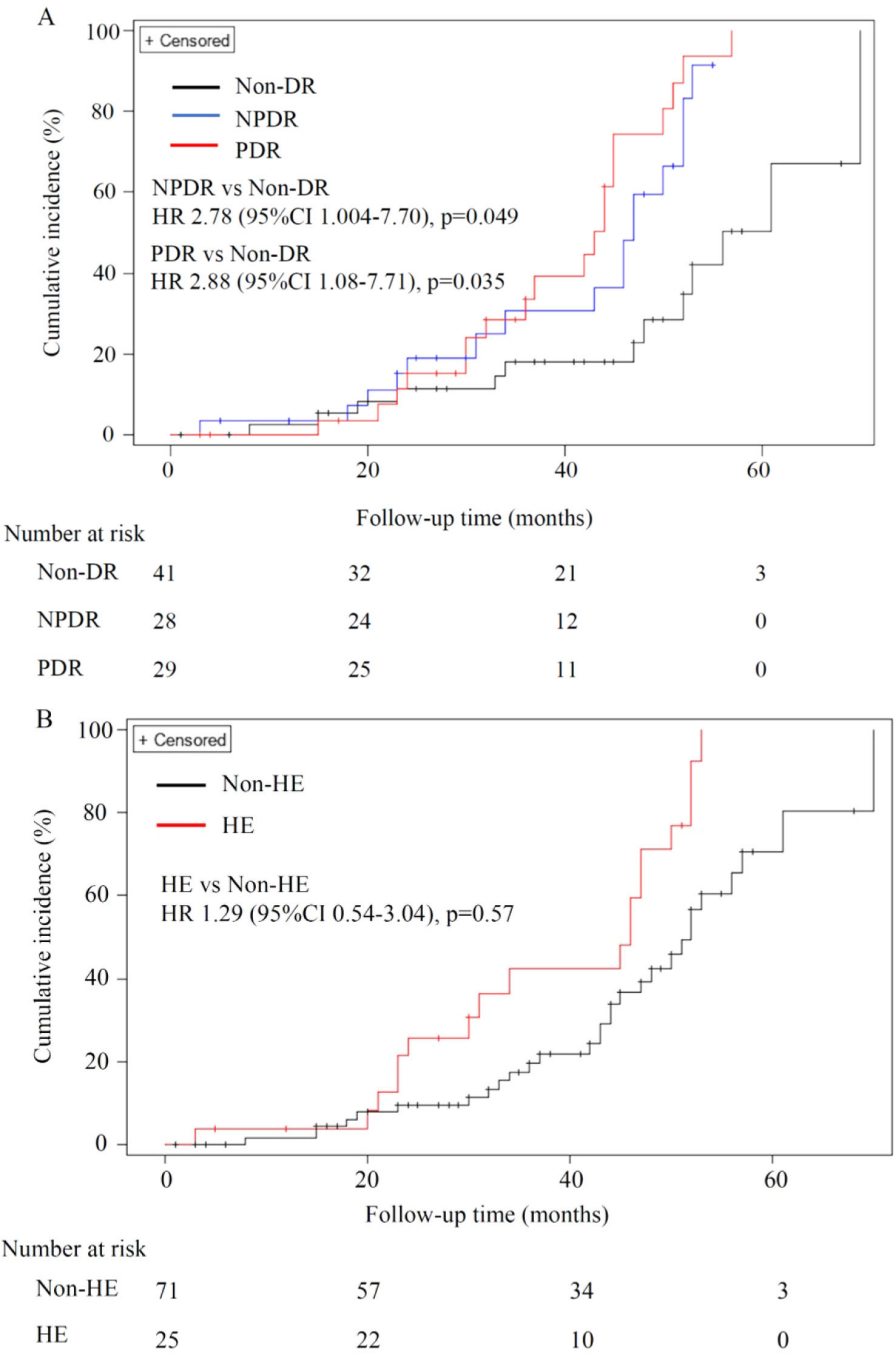
Impact of DR stage and HEs on the progression of DKD. **(A)** Participants with PDR and NPDR presented a nearly threefold greater risk (adjusted HR = 2.88; 95% CI: 1.08–7.71, p=0.035; adjusted HR = 2.78; 95% CI: 1.004–7.70, p=0.049 respectively) of eGFR decline (>15%) than did those without DR. **(B)** The presence of HEs was not independently associated with progression of DKD (adjusted HR = 1.29; 95% CI: 0.54–3.04, p=0.567).DKD, Diabetic kidney disease; DR, Diabetic retinopathy; NPDR, Non-proliferative diabetic retinopathy; PDR, Proliferative diabetic retinopathy; HEs, Hard exudates.

Cox regression analysis incorporating independent variables (p<0.1) such as the severity of DR or the presence of HEs, age, BMI, HbA1c level, LDL-C level, systolic pressure, eGFR at baseline, UACR stage at baseline, use of SGLT2 inhibitors, as well as potential confounders such as duration of DM, use of RAS inhibitors and GLP-1RAs revealed that the PDR (p=0.035) and NPDR (p=0.049) were significant and independent predictors of DKD progression. Specifically, participants with PDR, as well as NPDR presented a nearly threefold greater risk (adjusted HR = 2.88; 95% CI: 1.08–7.71, p=0.035; adjusted HR = 2.78; 95% CI: 1.004–7.70, p=0.049 respectively) than did those without DR (**Table 4**, **Figure 2A**). However, the presence of HEs was not independently associated with renal function deterioration according to Cox regression (adjusted HR = 1.29; 95% CI: 0.54–3.04, p=0.567). (**Table 4**, **Figure 2B**). Multivariate logistic regression analysis revealed similar findings concerning the predictive value of HEs for DKD progression (**Supplementary Table**).

**Table 4:** Cox regression analysis on the relationship between baseline DR stage and presence of HEs with the progression of DKD.

Variable	HR	95% CI	P value
Model 1			
DR stage			
Non-DR	reference		
NPDR	2.78	1.004-7.70	0.049^*^
PDR	2.88	1.08-7.71	0.035^*^
Age	1.04	1.003-1.09	0.034^*^
BMI	0.97	0.87-1.07	0.526
HbA1c (%)	1.11	0.94-1.33	0.225
Duration of DM (years)	1.03	0.98-1.08	0.209
LDL cholesterol (mmol/l)	1.04	0.73-1.48	0.828
Systolic pressure (mmHg)	1.01	1.00-1.02	0.185
eGFR stage baseline			
eGFR stage 1 (eGFR≥90ml/min/1.73m^2)	reference		
eGFR stage 2 (60ml/min/1.73m^2≤eGFR<90ml/min/1.73m^2)	2.75	0.82-9.20	0.101
eGFR stage 3 (eGFR<60ml/min/1.73m^2)	1.24	0.37-4.19	0.731
UACR stage baseline			
UACR stage 1(ACR<300mg/g)	reference		
UACR stage 2(ACR≥300mg/g)	4.27	1.54-11.82	0.005^*^
Use of RAS inhibitors	0.78	0.36-1.70	0.527
Use of SGLT2 inhibitors	0.19	0.05-0.72	0.015^*^
Use of GLP-1RAs	4.77	0.43-53.05	0.203
Model 2			
Presence of HEs			
Non-HEs	reference		
HEs	1.29	0.54-3.04	0.567
Age	1.03	0.99-1.07	0.181
BMI	0.96	0.86-1.07	0.471
HbA1c (%)	1.11	0.92-1.35	0.268
Duration of DM (years)	1.03	0.98-1.08	0.213
LDL cholesterol (mmol/l)	1.09	0.77-1.54	0.641
Systolic pressure (mmHg)	1.01	1.00-1.03	0.184
eGFR stage baseline			
eGFR stage 1 (eGFR≥90ml/min/1.73m^2)	reference		
eGFR stage 2 (60ml/min/1.73m^2≤eGFR<90ml/min/1.73m^2)	4.50	1.26-16.10	0.021^*^
eGFR stage 3 (eGFR<60ml/min/1.73m^2)	2.12	0.58-7.70	0.254
UACR stage baseline			
UACR stage 1(ACR<300mg/g)	reference		
UACR stage 2(ACR≥300mg/g)	4.13	1.50-11.43	0.006^*^
Use of RAS inhibitors	0.96	0.43-2.12	0.911
Use of SGLT2 inhibitors	0.21	0.06-0.71	0.012^*^
Use of GLP-1RAs	4.29	0.40-45.49	0.227

Variables with p value <0.10 and potential confounders such as duration of DM, use of RAS inhibitors, GLP-1RAs are included in the COX regression analysis. *p value <0.05.

DKD, Diabetic kidney disease; DM, Diabetic mellitus; DR, Diabetic retinopathy; NPDR, Non-proliferative diabetic retinopathy; PDR, Proliferative diabetic retinopathy; HEs, Hard exudates; BMI, Body Mass Index; HDL, LDL, Low-density lipoprotein cholesterol; eGFR, Estimated glomerular filtration rate; UACR, Urine albumin creatine ratio; RAS, Renin-Angiotensin System; SGLT2, Sodium-Glucose Linked Transporter 2; GLP-1RAs, Glucagon-Like Peptide-1 Receptor Agonists.

## Discussion

The present retrospective, observational cohort study demonstrated that the severity of DR was associated with the progression of DKD in patients with type 2 DM. Our findings revealed that patients with PDR and NPDR faced a risk of DKD progression that was nearly three times greater than that of patients without DR. However, the presence of HEs was not associated with DKD progression compared with the absence of HEs. Our results indicate that the severity of DR, not the presence of HEs, has clinical significance in the monitoring and management of DKD concerning eGFR decline.

As the leading cause of end-stage renal disease, DKD is thought to share key pathological pathways with DR, including chronic hyperglycemia, oxidative stress, and inflammation. This commonality suggests that the two disorders may engage in a vicious cycle, mutually accelerating each other’s progression (21–23). In a study conducted on patients with biopsy-confirmed DKD, DR was identified as an independent predictor for progression to end-stage renal disease (24). Furthermore, a retrospective multicenter study involving more than 800 patients over a 10-year follow-up period revealed an association between DR and an increased risk of kidney failure and the need for kidney replacement therapy (KRT) in patients with histologically confirmed DKD (22). These findings underscore the interconnected impact of these conditions on both renal and eye health.

In the present study, we defined the progression of DKD as an eGFR decrease (%)>15%, which is the median decrease in the eGFR over a mean follow-up period of approximately 35 months. While no universally accepted criteria for DKD progression exist, our definition aligns with those used in previous studies (25–27). Owing to the intrinsic pathological differences between NPDR and PDR, we categorized DR into these two groups rather than focusing solely on the presence of DR, as has been done mostly in earlier studies. We found that 57.1% of NPDR patients and 65.5% of PDR patients experienced DKD progression (p=0.024) (**Table 1**). Cox regression analysis indicated that PDR and NPDR were strongly and independently associated with DKD progression. Compared with patients in the absent DR group, participants with PDR, as well as NPDR had a nearly threefold greater risk (adjusted HR = 2.88; 95% CI: 1.08–7.71,p=0.035; adjusted HR = 2.78; 95% CI: 1.004–7.70, p=0.049 respectively). The results of our study were consistent with those of previous studies. A previous study (28) reported that moderate NPDR, severe NPDR and PDR were significantly associated with the progression of DKD. However, this earlier study primarily evaluated general type 2 DM patients and defined DKD progression as an eGFR decrease to less than 60 mL/min/1.73m^2^. In our study, we evaluated patients with DKD instead of general DM patients because renal function is more important in DKD patients with respect to patient management for clinicians.

HEs are lipid and protein deposits that form in the retina and are indicative of underlying vascular damage resulting from chronic hyperglycemia (17). Their formation closely mirrors that of urinary proteins, which arise from kidney vascular damage due to the same conditions. The pathophysiological link between HEs and renal outcomes can be attributed to shared microvascular alterations driven by chronic hyperglycemia, endothelial dysfunction, and associated inflammatory processes. Given this common mechanism, HEs may hold significant potential for monitoring microvascular complications in diabetic patients, particularly those already suffering from nephropathy. As a clinical marker of broader microvascular dysfunction in the retina, HEs are observable in both NPDR patients and PDR patients. They manifest as bright yellow deposits with sharp margins, appearing in clusters, spots, or rings, typically near areas of microaneurysms or hemorrhages within the retina. These deposits are easily recognizable in fundus images. Therefore, identifying the presence of HEs can address challenges posed by the reliance on ophthalmologists for detection.

However, few studies have focused on the relationship between HEs and DKD, leading to a question of their clinical importance in managing diabetes and its associated microvascular complications. In our study, we found that 72.0% of patients with HEs had an eGFR decrease (%) >15%, whereas 42.4% of patients without HEs experienced similar DKD progression. A significant difference in the rate of eGFR decline was observed between patients with and without HEs (p=0.011) (**Table 3**). However, Cox regression and multivariate logistic analyses revealed that the presence of HEs was not associated with DKD progression after adjustment for incorporating independent variables. The reason may be that HEs are lipids and proteins deposited from impaired retina vessels (17), which are resolvable in response to metabolic control (29). However, eGFR decline represents an irreversible structural damage of micro-vessels (30). Therefore, after adjustment for lipid profile and blood pressure etc., we found no significant difference in the association between HEs and DKD progression concerning eGFR decline. Appearance of HEs may reflect microvascular damage in retina, but disappearance of HEs does not guarantee improvement of microvascular condition. Thinking of that HEs are lipids and protein leaked from impaired retina vessels, they may be more strongly associated with the progression of UACR. To our knowledge, this is the first study to investigate the association between HEs and DKD progression. In the future, multicenter studies with larger cohorts and standardized follow-up including eGFR and UACR are needed to validate the causal relationships between the presence of HEs and DKD progression.

This study has several limitations. First, its retrospective cohort design precludes the determination of causality. Second, the single-center design and relatively small sample size of hospitalized patients may limit the generalizability of our findings to broader populations. Third, the follow-up duration was relatively short and heterogeneous among participants, resulting in a low frequency of outcome events. Fourth, DKD progression was defined exclusively by a decline in the eGFR. We were unable to incorporate the progression of albuminuria into this definition because of inconsistent measurements of the UACR during follow-up. Future prospective, multicenter studies with larger cohorts and standardized follow-up, as well as specific inclusion criteria regarding eGFR ranges are warranted to validate the relationships among DR severity, HEs, and renal functional decline. Such research would further clarify the clinical utility of these retinal markers in the management of patients with diabetes and nephropathy.

## Conclusions

In conclusion, this study demonstrated a significant association between DR severity and the progression of DKD. However, the presence of HEs was not associated with DKD progression after adjustment for incorporating independent variables. The stage of DR, not the presence of HEs, may serve as a clinical marker of renal microvascular conditions, providing clinicians with an easy and reliable way to evaluate renal risk through ophthalmic examinations. Furthermore, patients diagnosed with NPDR and PDR should undergo kidney function screening and receive early management to mitigate the progression of renal impairment. However, long-term prospective studies will be necessary to definitively establish causality between these retinal markers and the progression of renal function in individuals with DKD.

## Data Availability

The raw data supporting the conclusions of this article will be made available by the authors, without undue reservation.
