# Supplementary material for: Diabetic retinopathy severity is associated with renal function deterioration in patients with diabetic kidney disease: a retrospective cohort study
Source: Front Endocrinol (Lausanne). 2026 Feb 26;17:1757296. doi: 10.3389/fendo.2026.1757296 (PMC12980877; doi:10.3389/fendo.2026.1757296)
Supplement: Supplementary file 1 [file Table1.docx]

Supplementary Table. Multivariate logistic regression analysis on the relationship between baseline DR stage and presence of HEs with the progression of DKD

| Variable | OR | 95% CI | P |
| --- | --- | --- | --- |
| **Model 1** |  |  |  |
| DR stage |  |  |  |
| Non-DR | reference |  |  |
| NPDR | 4.52 | 0.83-24.58 | 0.081 |
| PDR | 10.48 | 1.47-74.83 | 0.019^*^ |
| Age | 1.09 | 1.01-1.18 | 0.027^*^ |
| BMI | 0.90 | 0.75-1.08 | 0.241 |
| HbA1c (%) | 1.75 | 1.15-2.65 | 0.009^*^ |
| Duration of DM (years) | 0.97 | 0.89-1.06 | 0.528 |
| LDL cholesterol (mmol/l) | 3.14 | 1.34-7.35 | 0.009^*^ |
| Systolic pressure (mmHg) | 0.998 | 0.97-1.03 | 0.887 |
| eGFR stage base |  |  |  |
| eGFR stage 1 (eGFR≥90ml/min/1.73m^2) | reference |  |  |
| eGFR stage 2 (60ml/min/1.73m^2≤eGFR＜90ml/min/1.73m^2) | 14.38 | 1.94-106.44 | 0.009^*^ |
| eGFR stage 3 (eGFR＜60ml/min/1.73m^2) | 8.94 | 1.30-61.27 | 0.026^*^ |
| UACR stage baseline |  |  |  |
| UACR stage 1(ACR<300mg/g) | reference |  |  |
| UACR stage 2(ACR≥300mg/g) | 1.25 | 0.27-5.70 | 0.775 |
| Use of RAS inhibitors | 6.85 | 0.99-47.63 | 0.051 |
| Use of SGLT2 inhibitors | 0.18 | 0.02-1.34 | 0.093 |
| Use of GLP-1RAs | 0.79 | 0.05-12.37 | 0.864 |
| Follow-up Time (months) | 1.08 | 1.03-1.14 | 0.002^*^ |
|  |  |  |  |
| **Model 2** |  |  |  |
| Presence of HEs |  |  |  |
| Non-HEs | reference |  |  |
| HEs | 2.97 | 1.03-1.13 | 0.159 |
| Age | 1.04 | 0.98-1.11 | 0.188 |
| BMI | 0.93 | 0.78-1.10 | 0.379 |
| HbA1c (%) | 1.47 | 1.02-2.12 | 0.040^*^ |
| Duration of DM (years) | 1.006 | 0.93-1.09 | 0.883 |
| LDL cholesterol (mmol/l) | 2.29 | 1.08-4.85 | 0.031^*^ |
| Systolic pressure (mmHg) | 1.006 | 0.98-1.03 | 0.684 |
| eGFR stage base |  |  |  |
| eGFR stage 1 (eGFR≥90ml/min/1.73m^2) | reference |  |  |
| eGFR stage 2 (60ml/min/1.73m^2≤eGFR＜90ml/min/1.73m^2) | 14.74 | 2.37-91.90 | 0.004^*^ |
| eGFR stage 3 (eGFR＜60ml/min/1.73m^2) | 12.26 | 1.86-80.90 | 0.009^*^ |
| UACR stage baseline |  |  |  |
| UACR stage 1(ACR<300mg/g) | reference |  |  |
| UACR stage 2(ACR≥300mg/g) | 1.40 | 0.32-6.15 | 0.658 |
| Use of RAS inhibitors | 4.17 | 0.78-22.29 | 0.095 |
| Use of SGLT2 inhibitors | 0.19 | 0.03-1.32 | 0.092 |
| Use of GLP-1RAs | 0.89 | 0.05-13.55 | 0.889 |
| Follow-up Time (months) | 1.08 | 1.03-1.13 | 0.003^*^ |

Variables with p value <0.10 and potential confounders such as duration of DM, use of RAS inhibitors, GLP-1RAs are included in the Multivariate logistic regression analysis. *p value <0.05

DKD: Diabetic kidney disease; DM: Diabetic mellitus; DR: Diabetic retinopathy; NPDR: Non-proliferative diabetic retinopathy; PDR: Proliferative diabetic retinopathy; HEs: Hard exudates; BMI: Body Mass Index; HDL: LDL: Low-density lipoprotein cholesterol，eGFR: Estimated glomerular filtration rate; UACR: Urine albumin creatine ratio; RAS: Renin-Angiotensin System; SGLT2: Sodium-Glucose Co-Transporter 2; GLP-1RAs: Glucagon-Like Peptide-1 Receptor Agonists
